# Crystal structure of (*Z*)-4-[1-(4-acetyl­anilino)ethyl­idene]-3-methyl-1-phenyl-1*H*-pyrazol-5(4*H*)-one

**DOI:** 10.1107/S2056989014026899

**Published:** 2015-01-01

**Authors:** Refaat M. Mahfouz, Zeynep Demircioğlu, Mohamed S. Abbady, Orhan Büyükgüngör

**Affiliations:** a Department of Chemistry, Faculty of Science, Assiut University, Assiut 71516, Egypt; b Department of Physics, Ondokuz Mayıs University, TR-55139 Samsun, Turkey

**Keywords:** crystal structure, Schiff bases, pyrazolone derivatives, keto–amine tautomeric form, hydrogen bonding, π–π stacking inter­actions

## Abstract

In the solid state, the title compound, C_20_H_19_N_3_O_2_, adopts the keto–amine tautomeric form, with the H atom attached to the N atom, which participates in an intra­molecular N—H⋯O hydrogen bond with an *S*(6) ring motif. The dihedral angles between the pyrazole ring and the phenyl and benzene rings are 3.69 (10) and 46.47 (9)°, respectively. In the crystal, mol­ecules are linked by weak C—H⋯O hydrogen bonds, generating *C*(16) chains propagating in [301]. Weak aromatic π–π stacking inter­actions [centroid–centroid distances = 3.6123 (10) and 3.6665 (10) Å] link the chains into a three-dimensional network.

## Abstract 2

In the solid state, the title compound, adopts the keto–amine tautomeric form, with the H atom attached to the N atom, which participates in an intra­molecular N—H⋯O hydrogen bond with an *S*(6) ring motif. In the crystal, mol­ecules are linked by weak C—H⋯O hydrogen bonds to generate *C*(16) chains propagating in the [301] direction.

## Chemical context   

The chemistry of pyrazolone derivatives has attracted much attention because of their inter­esting structural properties and applications in diverse areas. Pyrazolone derivatives are also used as starting materials for the synthesis of biologically active compounds. Ethyl­idene species are of inter­est for this reaction system because they are a secondary C_2_ reaction inter­mediate, after ethyl species, expected from ethane by cleavage of two C—H bonds at the same carbon atom (Brooks *et al.*, 2011 ▸).

Schiff base compounds have received considerable attention for many years, primarily due to their importance in the development of coordination chemistry related to magnetism (Weber *et al.*, 2007 ▸), catalysis (Chen *et al.*, 2008 ▸) and biological processes (May *et al.*, 2004 ▸). In general, *O*-hy­droxy Schiff bases exhibit two possible tautomeric forms, the enol–imine and keto–amine forms. Depending on the tautomers, two types of intra­molecular hydrogen bonds are possible: O—H⋯N in the enol–imine and N—H⋯O in the keto–amine form. Schiff bases derived from acyl pyrazones and aromatic amines have been prepared as anti­microbial agents (Parmar *et al.*, 2015 ▸) and also as ligands for the formation of metal-ion complexes (Jayarajan *et al.*, 2010 ▸; Moorjani *et al.*, 2010 ▸). A compound similar to the title compound, 5-methyl-2-phenyl-4-{1-[(pyridin-2-ylmeth­yl)-amino]-ethyl­idene}-2,4-di­hydro-pyrazol-3-one derived from acyl pyrazolone and aliphatic amine was reported to possesses the amino-one structure (Amarasekara *et al.*, 2009 ▸).
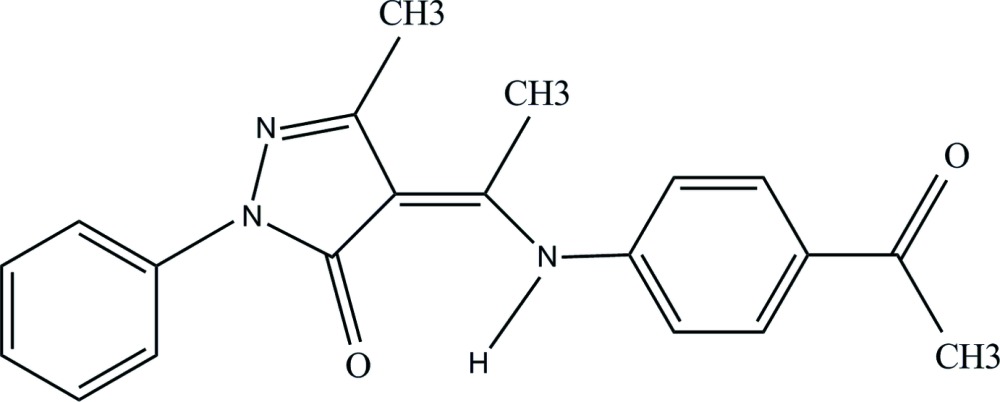


## Structural commentary   

**Figure 1:**
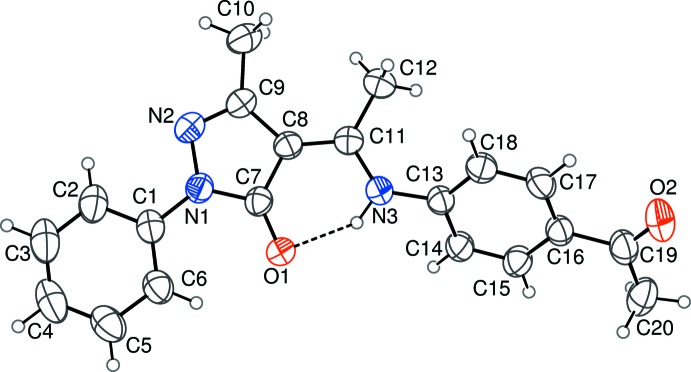
An *ORTEP* view of title compound, showing 30% probability displacement ellipsoids. The dashed line shows the intra­molecular N—H⋯O hydrogen bond.

In the title compound (Fig. 1 ▸) the bond lengths indicate double-bond character for the C7=O1 [1.2472 (19) Å [and C8=C11 [1.389 (2) Å] bonds and single-bond character for the C11—N3 [1.339 (2) Å] and N3—C13 [1.413 (2) Å] bonds. Furthermore, the H1 atom was found to be located on atom N3, confirming that the title compound exists in the keto–amine form in the solid state.

An intra­molecular N3—H3*A*⋯O1 hydrogen bond is observed (Table 1 ▸, Fig. 1 ▸). This inter­action generates an *S*(6) ring motif. The 4-acetyl­phenyl­amino ethyl­idene and phenyl pyrazol groups of the mol­ecule are nearly planar, with r.m.s. deviations from the mean plane of 0.0430 and 0.0256 Å, respectively. The dihedral angle between these two groups is 47.81 (3)°. The dihedral angles between the pyrazole ring and the phenyl and benzene rings are 3.69 (10) and 46.47 (9)°, respectively. Similar results were observed in *N*-[(3-methyl-5-oxo-1-phenyl-4,5-di­hydro-1*H*-pyrazol-4-yl­idene)(phen­yl)meth­­yl]glycine ethyl ester (Zhang *et al.*, 2004 ▸), ethyl 2-{[(1*Z*)-(3-methyl-5-oxo-1-phenyl-4,5-di­hydro-1*H*-pyrazol-4-yl­idene)(*p*-tol­yl)meth­yl]amino}-3-phenyl­propano­ate (Zhang *et al.*, 2010 ▸) and 4-{[3,4-di­hydro-5-methyl-3-oxo-2-phenyl-2*H*-pyrazol-4-yl­idene](phen­yl)methyl­amino}-1,5-dimethyl-2-phenyl-1*H*-pyrazol-3(2*H*)-one (Wang *et al.*, 2003 ▸).

## Supra­molecular features   

**Figure 2:**
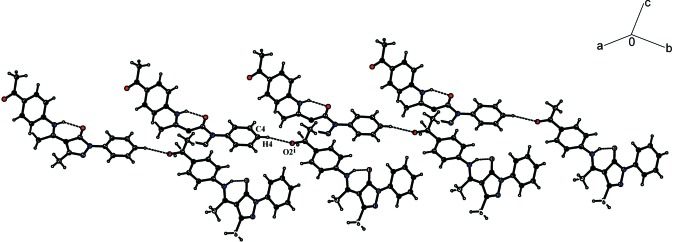
A packing diagram for title compound, showing the inter­molecular C—H⋯O and intra­molecular N—H⋯O hydrogen bonds. [Symmetry code: (i) 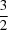 + *x*, 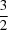 − *y*, 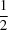 + *z*.]

**Figure 3:**
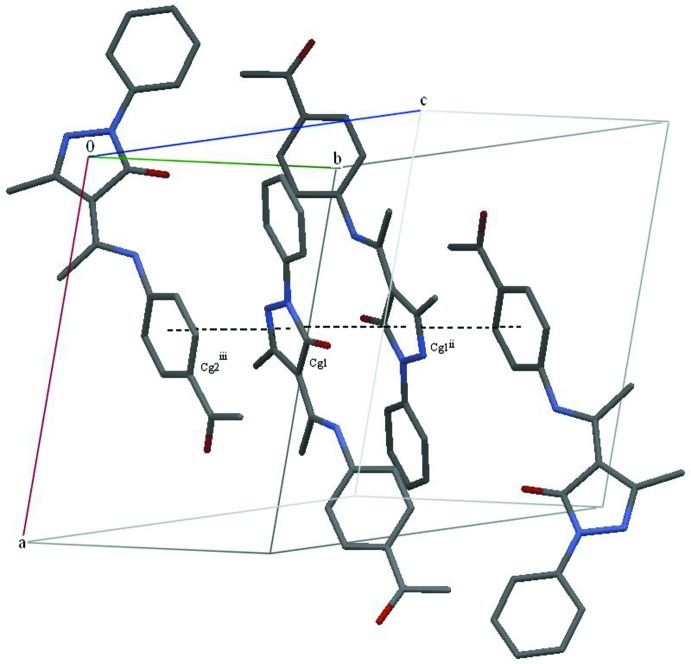
A packing diagram for title compound showing the π–π stacking inter­actions (dashed lines). H atoms not involved in hydrogen bonding have been omitted for clarity. *Cg*1 and *Cg2* are the centroids of the pyrozolone and C13–C18 rings, respectively. [Symmetry codes: (ii) 1 − *x*, 1 − *y*, 1 − *z*; (iii) 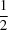 + *x*, 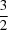 − *y*, 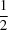 + *z*.]

In the crystal, the mol­ecules are linked by C4—H4⋯O2 hydrogen bonds (Fig. 2 ▸, Table 1 ▸). The chains formed by these bonds along the *c*-axis direction are connected by two weak π–π stacking inter­actions [*Cg*1⋯*Cg*1(1 − *x*, 1 − *y*, 1 − *z*) = 3.6123 (10) and *Cg*1⋯*Cg*2(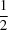 + *x*, 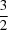 − *y*, 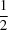 + *z*) = 3.6665 (10) Å; *Cg*1 and *Cg*2 are the centroids of the C7–C9/N1,N2 and C13–C18 rings, respectively], forming a three-dimensional network (Fig. 3 ▸).

## Synthesis and crystallization   

The title compound was obtained by refluxing equimolar qu­anti­ties of 4-acetyl-3-methyl-1-phenyl-2-pyrazolin-5-one and 4-amino­aceto­phenone (10 mmol) in ethanol for 2 h. On cooling, the yellow precipitate was collected by filtration and recrystallized from an ethanol–dioxan solvent mixture as yellow slabs. Yield (73%); m.p. 439–441; IR (KBr) ν = 3450, 3350, 3300 (NH_2_, NH), 1676,1628 (C=O, *s*) cm^−1^; MS, *m*/*z* = 333.8. Calculated for C_20_H_19_N_3_O_2_: C, 72.05; H, 5.74; N, 12.60. Found: C, 72.20; H, 5.62; N, 12.78%.

## Refinement   

Crystal data, data collection and structure refinement details are summarized in Table 2 ▸. The H atom bonded to the N atom was located in a difference Fourier map and was refined freely. All other H atoms were refined using a riding model with *d*(C—H) = 0.93 Å (*U*_iso_=1.2*U*_eq_ of the parent atom) for aromatic C atoms and *d*(C—H) = 0.96 Å (*U*_iso_=1.5*U*_eq_ of the parent atom) for methyl C atoms.

## Supplementary Material

Crystal structure: contains datablock(s) I, global. DOI: 10.1107/S2056989014026899/hb7330sup1.cif

Structure factors: contains datablock(s) I. DOI: 10.1107/S2056989014026899/hb7330Isup2.hkl

Supporting information file. DOI: 10.1107/S2056989014026899/hb7330Isup3.cml

CCDC reference: 1038012

Additional supporting information:  crystallographic information; 3D view; checkCIF report

## Figures and Tables

**Table 1:** Hydrogen-bond geometry (, )

*D*H*A*	*D*H	H*A*	*D* *A*	*D*H*A*
N3H3*A*O1	0.90(2)	1.88(2)	2.6527(18)	144(2)
C4H4O2^i^	0.93	2.57	3.403(2)	150

Symmetry code: (i) 
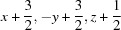
.

**Table 2:** Experimental details

Crystal data	
Chemical formula	C_20_H_19_N_3_O_2_
*M*_r_	333.38
Crystal system, space group	Monoclinic, *P*2_1_/*n*
Temperature (K)	296
*a*, *b*, *c* ()	11.8549(4), 11.6070(5), 13.1591(5)
()	107.425(3)
*V* (^3^)	1727.60(12)
*Z*	4
Radiation type	Mo *K*
(mm^1^)	0.09
Crystal size (mm)	0.80 0.57 0.10

Data collection	
Diffractometer	Stoe *IPDS* 2
Absorption correction	Integration (*X-RED32*; Stoe Cie, 2002 ▸)
*T*_min_, *T*_max_	0.935, 0.991
No. of measured, independent and observed [*I* > 2(*I*)] reflections	25592, 3584, 2772
*R*_int_	0.056
(sin /)_max_ (^1^)	0.628

Refinement	
*R*[*F* ^2^ > 2(*F* ^2^)], *wR*(*F* ^2^), *S*	0.050, 0.126, 1.07
No. of reflections	3584
No. of parameters	231
No. of restraints	1
H-atom treatment	H atoms treated by a mixture of independent and constrained refinement
_max_, _min_ (e ^3^)	0.17, 0.16

Computer programs: *X-AREA* and *X-RED32* (Stoe Cie, 2002 ▸), *SHELXS97* and *SHELXL97* (Sheldrick, 2008 ▸), *ORTEP-3 for Windows* and *WinGX* (Farrugia, 2012 ▸) and *PLATON* (Spek, 2009 ▸).

## supplementary crystallographic information

### Crystal data

**Table:**

C_20_H_19_N_3_O_2_	*F*(000) = 704
*M**_r_* = 333.38	*D*_x_ = 1.282 Mg m^−^^3^
Monoclinic, *P*2_1_/*n*	Mo *K*α radiation, λ = 0.71073 Å
*a* = 11.8549 (4) Å	Cell parameters from 3705 reflections
*b* = 11.6070 (5) Å	θ = 2.4–26.7°
*c* = 13.1591 (5) Å	µ = 0.09 mm^−^^1^
β = 107.425 (3)°	*T* = 296 K
*V* = 1727.60 (12) Å^3^	Slab, yellow
*Z* = 4	0.80 × 0.57 × 0.10 mm

### Data collection

**Table:**

Stoe IPDS 2 diffractometer	2772 reflections with *I* > 2σ(*I*)
Radiation source: fine-focus sealed tube	*R*_int_ = 0.056
ω–scan rotation method	θ_max_ = 26.5°, θ_min_ = 2.4°
Absorption correction: integration (*X-RED32*; Stoe & Cie, 2002)	*h* = −14→14
*T*_min_ = 0.935, *T*_max_ = 0.991	*k* = −14→14
25592 measured reflections	*l* = −16→16
3584 independent reflections	

### Refinement

**Table:**

Refinement on *F*^2^	Primary atom site location: structure-invariant direct methods
Least-squares matrix: full	Secondary atom site location: difference Fourier map
*R*[*F*^2^ > 2σ(*F*^2^)] = 0.050	Hydrogen site location: mixed
*wR*(*F*^2^) = 0.126	H atoms treated by a mixture of independent and constrained refinement
*S* = 1.07	*w* = 1/[σ^2^(*F*_o_^2^) + (0.0592*P*)^2^ + 0.1897*P*] where *P* = (*F*_o_^2^ + 2*F*_c_^2^)/3
3584 reflections	(Δ/σ)_max_ < 0.001
231 parameters	Δρ_max_ = 0.17 e Å^−^^3^
1 restraint	Δρ_min_ = −0.16 e Å^−^^3^

### Special details

**Table:**

Geometry. All s.u.'s (except the s.u. in the dihedral angle between two l.s. planes) are estimated using the full covariance matrix. The cell s.u.'s are taken into account individually in the estimation of s.u.'s in distances, angles and torsion angles; correlations between s.u.'s in cell parameters are only used when they are defined by crystal symmetry. An approximate (isotropic) treatment of cell s.u.'s is used for estimating s.u.'s involving l.s. planes.

### Fractional atomic coordinates and isotropic or equivalent isotropic displacement parameters (Å^2^)

**Table:**

	*x*	*y*	*z*	*U*_iso_*/*U*_eq_	
C1	0.68663 (14)	0.70961 (15)	0.55951 (13)	0.0594 (4)	
C2	0.78901 (16)	0.70082 (19)	0.64551 (16)	0.0771 (5)	
H2	0.7865	0.6647	0.7079	0.093*	
C3	0.89330 (18)	0.7453 (2)	0.6381 (2)	0.0931 (7)	
H3	0.9614	0.7388	0.6957	0.112*	
C4	0.89876 (19)	0.7993 (2)	0.5472 (2)	0.0923 (7)	
H4	0.9701	0.8287	0.5427	0.111*	
C5	0.79784 (19)	0.8096 (2)	0.46270 (19)	0.0838 (6)	
H5	0.8011	0.8472	0.4012	0.101*	
C6	0.69112 (16)	0.76487 (17)	0.46756 (16)	0.0698 (5)	
H6	0.6234	0.7719	0.4097	0.084*	
C7	0.47116 (14)	0.65691 (14)	0.49191 (12)	0.0542 (4)	
C8	0.39461 (14)	0.60235 (13)	0.54463 (12)	0.0530 (4)	
C9	0.46974 (15)	0.57875 (15)	0.65068 (12)	0.0583 (4)	
C10	0.4410 (2)	0.5216 (2)	0.74124 (14)	0.0825 (6)	
H10A	0.4126	0.4450	0.7208	0.099*	
H10B	0.3811	0.5651	0.7598	0.099*	
H10C	0.5107	0.5179	0.8015	0.099*	
C11	0.27542 (14)	0.58305 (14)	0.49366 (12)	0.0536 (4)	
C12	0.19337 (16)	0.53059 (17)	0.54741 (14)	0.0685 (5)	
H12A	0.1621	0.5898	0.5822	0.103*	
H12B	0.2356	0.4753	0.5992	0.103*	
H12C	0.1296	0.4929	0.4954	0.103*	
C13	0.11905 (14)	0.61084 (14)	0.31950 (12)	0.0545 (4)	
C14	0.10632 (15)	0.57899 (16)	0.21567 (13)	0.0633 (4)	
H14	0.1718	0.5538	0.1968	0.076*	
C15	−0.00252 (15)	0.58422 (17)	0.13969 (14)	0.0661 (5)	
H15	−0.0096	0.5630	0.0699	0.079*	
C16	−0.10156 (14)	0.62055 (15)	0.16568 (13)	0.0595 (4)	
C17	−0.08768 (15)	0.65196 (16)	0.27021 (15)	0.0663 (5)	
H17	−0.1534	0.6762	0.2892	0.080*	
C18	0.02076 (15)	0.64829 (16)	0.34677 (14)	0.0647 (4)	
H18	0.0281	0.6708	0.4163	0.078*	
C19	−0.22040 (16)	0.63304 (18)	0.08467 (17)	0.0739 (5)	
C20	−0.23519 (19)	0.5992 (2)	−0.02808 (16)	0.0863 (6)	
H20A	−0.2151	0.5193	−0.0307	0.104*	
H20B	−0.1841	0.6453	−0.0560	0.104*	
H20C	−0.3158	0.6111	−0.0700	0.104*	
N1	0.57996 (12)	0.66251 (12)	0.56781 (10)	0.0576 (3)	
N2	0.57755 (13)	0.61356 (13)	0.66442 (11)	0.0639 (4)	
N3	0.23507 (12)	0.61234 (13)	0.39067 (11)	0.0586 (4)	
O1	0.44674 (10)	0.69356 (12)	0.39873 (9)	0.0673 (3)	
O2	−0.30253 (13)	0.67134 (17)	0.11080 (14)	0.1087 (6)	
H3A	0.2925 (16)	0.6339 (18)	0.3635 (15)	0.086 (6)*	

### Atomic displacement parameters (Å^2^)

**Table:**

	*U*^11^	*U*^22^	*U*^33^	*U*^12^	*U*^13^	*U*^23^
C1	0.0516 (9)	0.0619 (10)	0.0649 (10)	−0.0006 (7)	0.0176 (7)	−0.0177 (8)
C2	0.0589 (10)	0.0909 (14)	0.0741 (12)	−0.0009 (10)	0.0089 (9)	−0.0204 (10)
C3	0.0577 (11)	0.1136 (18)	0.1009 (17)	−0.0079 (11)	0.0128 (11)	−0.0380 (15)
C4	0.0619 (12)	0.1057 (17)	0.1176 (18)	−0.0214 (11)	0.0397 (12)	−0.0490 (15)
C5	0.0779 (13)	0.0907 (15)	0.0948 (15)	−0.0168 (11)	0.0439 (12)	−0.0234 (12)
C6	0.0608 (10)	0.0796 (12)	0.0721 (11)	−0.0067 (9)	0.0247 (9)	−0.0135 (9)
C7	0.0521 (8)	0.0580 (9)	0.0512 (8)	0.0024 (7)	0.0136 (7)	−0.0054 (7)
C8	0.0568 (9)	0.0529 (9)	0.0501 (8)	0.0012 (7)	0.0170 (7)	−0.0024 (7)
C9	0.0649 (10)	0.0575 (9)	0.0504 (8)	0.0038 (8)	0.0140 (7)	−0.0007 (7)
C10	0.0912 (14)	0.0940 (15)	0.0590 (10)	−0.0028 (12)	0.0177 (10)	0.0137 (10)
C11	0.0579 (9)	0.0505 (9)	0.0537 (8)	0.0007 (7)	0.0186 (7)	−0.0032 (7)
C12	0.0693 (11)	0.0700 (11)	0.0697 (11)	−0.0055 (9)	0.0263 (9)	0.0063 (9)
C13	0.0499 (8)	0.0561 (9)	0.0568 (9)	−0.0027 (7)	0.0148 (7)	0.0001 (7)
C14	0.0529 (9)	0.0800 (12)	0.0587 (9)	0.0079 (8)	0.0195 (7)	−0.0031 (8)
C15	0.0619 (10)	0.0783 (12)	0.0554 (9)	0.0041 (9)	0.0133 (8)	−0.0057 (8)
C16	0.0504 (9)	0.0597 (10)	0.0663 (10)	−0.0039 (7)	0.0145 (7)	0.0045 (8)
C17	0.0525 (9)	0.0752 (12)	0.0760 (11)	0.0051 (8)	0.0267 (8)	0.0036 (9)
C18	0.0620 (10)	0.0753 (12)	0.0602 (9)	0.0041 (9)	0.0233 (8)	−0.0053 (8)
C19	0.0530 (10)	0.0735 (12)	0.0898 (13)	−0.0073 (9)	0.0133 (9)	0.0103 (10)
C20	0.0737 (13)	0.0826 (14)	0.0827 (13)	−0.0088 (11)	−0.0069 (10)	0.0038 (11)
N1	0.0527 (7)	0.0649 (8)	0.0527 (7)	0.0008 (6)	0.0120 (6)	−0.0036 (6)
N2	0.0657 (9)	0.0692 (9)	0.0519 (7)	0.0039 (7)	0.0104 (6)	0.0015 (6)
N3	0.0496 (7)	0.0718 (9)	0.0546 (8)	−0.0041 (6)	0.0158 (6)	−0.0003 (6)
O1	0.0583 (7)	0.0915 (9)	0.0505 (6)	−0.0052 (6)	0.0139 (5)	0.0071 (6)
O2	0.0523 (8)	0.1516 (16)	0.1176 (13)	0.0109 (9)	0.0185 (8)	0.0077 (11)

### Geometric parameters (Å, º)

**Table:**

C1—C6	1.385 (3)	C11—C12	1.493 (2)
C1—C2	1.393 (2)	C12—H12A	0.9600
C1—N1	1.412 (2)	C12—H12B	0.9600
C2—C3	1.370 (3)	C12—H12C	0.9600
C2—H2	0.9300	C13—C14	1.380 (2)
C3—C4	1.370 (4)	C13—C18	1.388 (2)
C3—H3	0.9300	C13—N3	1.413 (2)
C4—C5	1.373 (3)	C14—C15	1.377 (2)
C4—H4	0.9300	C14—H14	0.9300
C5—C6	1.387 (3)	C15—C16	1.383 (2)
C5—H5	0.9300	C15—H15	0.9300
C6—H6	0.9300	C16—C17	1.384 (2)
C7—O1	1.2472 (19)	C16—C19	1.498 (2)
C7—N1	1.376 (2)	C17—C18	1.376 (2)
C7—C8	1.443 (2)	C17—H17	0.9300
C8—C11	1.389 (2)	C18—H18	0.9300
C8—C9	1.439 (2)	C19—O2	1.210 (2)
C9—N2	1.300 (2)	C19—C20	1.494 (3)
C9—C10	1.490 (2)	C20—H20A	0.9600
C10—H10A	0.9600	C20—H20B	0.9600
C10—H10B	0.9600	C20—H20C	0.9600
C10—H10C	0.9600	N1—N2	1.4006 (19)
C11—N3	1.339 (2)	N3—H3A	0.895 (15)
			
C6—C1—C2	119.44 (17)	H12A—C12—H12B	109.5
C6—C1—N1	121.12 (15)	C11—C12—H12C	109.5
C2—C1—N1	119.44 (17)	H12A—C12—H12C	109.5
C3—C2—C1	120.0 (2)	H12B—C12—H12C	109.5
C3—C2—H2	120.0	C14—C13—C18	119.26 (15)
C1—C2—H2	120.0	C14—C13—N3	117.11 (14)
C4—C3—C2	120.9 (2)	C18—C13—N3	123.42 (15)
C4—C3—H3	119.5	C15—C14—C13	120.55 (15)
C2—C3—H3	119.5	C15—C14—H14	119.7
C3—C4—C5	119.3 (2)	C13—C14—H14	119.7
C3—C4—H4	120.3	C14—C15—C16	120.94 (16)
C5—C4—H4	120.3	C14—C15—H15	119.5
C4—C5—C6	121.1 (2)	C16—C15—H15	119.5
C4—C5—H5	119.5	C15—C16—C17	117.95 (15)
C6—C5—H5	119.5	C15—C16—C19	122.73 (16)
C1—C6—C5	119.22 (19)	C17—C16—C19	119.24 (16)
C1—C6—H6	120.4	C18—C17—C16	121.75 (16)
C5—C6—H6	120.4	C18—C17—H17	119.1
O1—C7—N1	126.05 (15)	C16—C17—H17	119.1
O1—C7—C8	128.90 (14)	C17—C18—C13	119.55 (16)
N1—C7—C8	105.04 (13)	C17—C18—H18	120.2
C11—C8—C9	133.02 (15)	C13—C18—H18	120.2
C11—C8—C7	122.26 (14)	O2—C19—C20	120.93 (18)
C9—C8—C7	104.71 (14)	O2—C19—C16	119.9 (2)
N2—C9—C8	111.89 (15)	C20—C19—C16	119.16 (18)
N2—C9—C10	118.56 (15)	C19—C20—H20A	109.5
C8—C9—C10	129.55 (16)	C19—C20—H20B	109.5
C9—C10—H10A	109.5	H20A—C20—H20B	109.5
C9—C10—H10B	109.5	C19—C20—H20C	109.5
H10A—C10—H10B	109.5	H20A—C20—H20C	109.5
C9—C10—H10C	109.5	H20B—C20—H20C	109.5
H10A—C10—H10C	109.5	C7—N1—N2	111.72 (13)
H10B—C10—H10C	109.5	C7—N1—C1	128.96 (14)
N3—C11—C8	116.82 (14)	N2—N1—C1	119.31 (13)
N3—C11—C12	119.81 (15)	C9—N2—N1	106.63 (13)
C8—C11—C12	123.36 (14)	C11—N3—C13	130.49 (14)
C11—C12—H12A	109.5	C11—N3—H3A	113.1 (13)
C11—C12—H12B	109.5	C13—N3—H3A	116.4 (13)
			
C6—C1—C2—C3	−0.8 (3)	C15—C16—C17—C18	0.4 (3)
N1—C1—C2—C3	179.45 (18)	C19—C16—C17—C18	−176.39 (17)
C1—C2—C3—C4	0.3 (3)	C16—C17—C18—C13	−0.8 (3)
C2—C3—C4—C5	0.5 (3)	C14—C13—C18—C17	0.6 (3)
C3—C4—C5—C6	−0.9 (3)	N3—C13—C18—C17	175.11 (17)
C2—C1—C6—C5	0.5 (3)	C15—C16—C19—O2	−175.8 (2)
N1—C1—C6—C5	−179.81 (16)	C17—C16—C19—O2	0.7 (3)
C4—C5—C6—C1	0.4 (3)	C15—C16—C19—C20	3.4 (3)
O1—C7—C8—C11	−0.1 (3)	C17—C16—C19—C20	179.97 (18)
N1—C7—C8—C11	178.89 (14)	O1—C7—N1—N2	179.96 (15)
O1—C7—C8—C9	−179.84 (17)	C8—C7—N1—N2	0.89 (17)
N1—C7—C8—C9	−0.80 (16)	O1—C7—N1—C1	0.2 (3)
C11—C8—C9—N2	−179.15 (17)	C8—C7—N1—C1	−178.83 (15)
C7—C8—C9—N2	0.49 (19)	C6—C1—N1—C7	3.6 (3)
C11—C8—C9—C10	1.4 (3)	C2—C1—N1—C7	−176.73 (16)
C7—C8—C9—C10	−178.95 (18)	C6—C1—N1—N2	−176.12 (16)
C9—C8—C11—N3	−177.17 (17)	C2—C1—N1—N2	3.6 (2)
C7—C8—C11—N3	3.2 (2)	C8—C9—N2—N1	0.04 (19)
C9—C8—C11—C12	1.8 (3)	C10—C9—N2—N1	179.55 (16)
C7—C8—C11—C12	−177.78 (16)	C7—N1—N2—C9	−0.60 (19)
C18—C13—C14—C15	0.0 (3)	C1—N1—N2—C9	179.15 (14)
N3—C13—C14—C15	−174.80 (16)	C8—C11—N3—C13	−175.30 (16)
C13—C14—C15—C16	−0.5 (3)	C12—C11—N3—C13	5.7 (3)
C14—C15—C16—C17	0.3 (3)	C14—C13—N3—C11	−142.71 (18)
C14—C15—C16—C19	176.95 (17)	C18—C13—N3—C11	42.7 (3)

### Hydrogen-bond geometry (Å, º)

**Table:**

*D*—H···*A*	*D*—H	H···*A*	*D*···*A*	*D*—H···*A*
N3—H3*A*···O1	0.90 (2)	1.88 (2)	2.6527 (18)	144 (2)
C4—H4···O2^i^	0.93	2.57	3.403 (2)	150

Symmetry code: (i) *x*+3/2, −*y*+3/2, *z*+1/2.
